# Analysis of GNSS, Hydroacoustic and Optoelectronic Data Integration Methods Used in Hydrography

**DOI:** 10.3390/s21237831

**Published:** 2021-11-25

**Authors:** Oktawia Lewicka, Mariusz Specht, Andrzej Stateczny, Cezary Specht, David Brčić, Alen Jugović, Szymon Widźgowski, Marta Wiśniewska

**Affiliations:** 1 Department of Geodesy and Oceanography, Gdynia Maritime University, Morska 81-87, 81-225 Gdynia, Poland; o.lewicka@wn.umg.edu.pl (O.L.); c.specht@wn.umg.edu.pl (C.S.); 2 Marine Technology Ltd., Wiktora Roszczynialskiego 4-6, 81-521 Gdynia, Poland; m.specht@marinetechnology.pl (M.S.); s.widzgowski@marinetechnology.pl (S.W.); m.wisniewska@marinetechnology.pl (M.W.); 3 Department of Geodesy, Gdańsk University of Technology, Gabriela Narutowicza 11-12, 80-233 Gdańsk, Poland; 4 Faculty of Maritime Studies, University of Rijeka, Studentska ulica 2, 51000 Rijeka, Croatia; david.brcic@pfri.uniri.hr (D.B.); alen.jugovic@pfri.uniri.hr (A.J.)

**Keywords:** data integration, Global Navigation Satellite System (GNSS), hydroacoustic methods, optoelectronic methods, hydrographic surveys

## Abstract

The integration of geospatial data in hydrography, performed using different measurement systems, involves combining several study results to provide a comprehensive analysis. Each of the hydroacoustic and optoelectronic systems is characterised by a different spatial reference system and the method for technical implementation of the measurement. Therefore, the integration of hydrographic data requires that problems in selected fields of electronics, geodesy and physics (acoustics and optics) be solved. The aim of this review is to present selected fusion methods applying the data derived from Global Navigation Satellite System (GNSS), Real Time Kinematic (RTK) measurements, hydrographic surveys, a photogrammetric pass using unmanned vehicles and Terrestrial Laser Scanning (TLS) and compare their accuracy. An additional goal is the evalution of data integration methods according to the International Hydrographic Organization (IHO) S-44 standard. The publication is supplemented by implementation examples of the integration of geospatial data in the Geographic Information System (GIS). The methods described indicate the lack of a uniform methodology for data fusion due to differences in both the spatial reference systems and the techniques used. However, the integration of hydroacoustic and optoelectronic data allows for high accuracy geospatial data to be obtained. This is confirmed by the methods cited, in which the accuracy of integrated geospatial data was in the order of several centimetres.

## 1. Introduction

The marine coastal zone is the most dynamic (in terms of geomorphological changes) area on the Earth. It includes the interface of the land and the sea and is under constant impact of the atmosphere, hydrosphere and intensive human activities [1,2]. Due to the intensive human use of coastal areas as well as the results of climate change, increased precipitation, rising sea levels and storm surges have occurred in this area [3]. Therefore, coastal zone monitoring, including bathymetry and coastal topography should be carried out.

**Figure 1:**
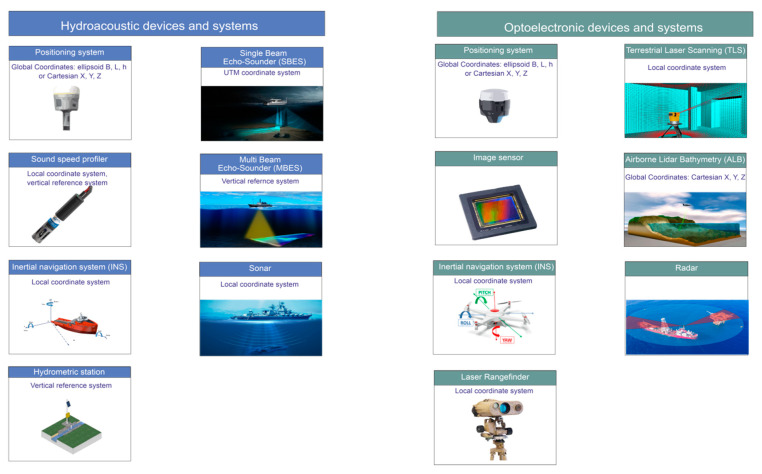
A diagram presenting hydroacoustic (blue colour) and optoelectronic (green colour) devices and systems.

In view of the rapid development of the devices and systems applied in hydrography, bathymetric surveys have been conducted using hydroacoustic methods [4]. The operation of hydroacoustic devices [5] is based on the phenomenon of acoustic location. The system sends out a high frequency sound wave into the water and then records the vibrations of the wave reflected off the bottom. The time and velocity of the sent sound wave enable the calculation of the depth at a selected bottom point. Bathymetric surveys use devices and systems with different spatial reference systems, including the following [6] (Figure 1):

- A positioning system is used to determine the coordinates of the vessel’s position. In hydrography, the most commonly applied positioning system is a Differential Global Positioning System (DGPS) or a Real Time Kinematic (RTK) receiver [7]. It determines the position coordinates that are presented in curvilinear coordinates (B, L, h) in relation to the reference ellipsoid used or in Cartesian coordinates (X, Y, Z);
- A sound velocity probe determines the sound velocity in water. The propagation of sound waves in water can be measured directly with a Sound Velocity Profile (SVP) or indirectly using a Conductivity, Temperature, Depth (CTD) sensor which measures the conductivity, hydrostatic pressure and temperature in seawater and then, based on these physicochemical variables, determines the sound velocity, as well as the seawater density and salinity [8]. A measurement of a sound wave in a vertical distribution is expressed in a vertical datum, while in a water column, it is expressed in a local datum associated with the sensor location;
- An Inertial Navigation System (INS) records the pitch, roll and yaw angles (RPY angles) of a vessel. It enables the determination of the vehicle’s orientation based on the knowledge of the RPY angles in the presence of disturbances due to waves and wind. Devices equipped with sensors of this type measure linear accelerations and rotation angles in three planes (X, Y, Z) in relation to a specified local system. Linear accelerations are determined by applying accelerometers, while the RPY angles are measured using a gyroscope;
- A hydrometric station records and collects data concerning the water quantity status on a lake, reservoir or river. An instrument used to measure water levels is a staff gauge, while the device used for the same purpose is a tide gauge. Some hydrometric stations are equipped with a telemetric function which enables automated data transfer, i.e., a General Packet Radio Service (GPRS) or radio modem. Information on water levels during bathymetric surveys enables the expression of the measured depths in relation to a pre-determined vertical datum. Changes in water levels need to be recorded and included during the hydrographic data processing;
- A Single Beam EchoSounder (SBES) is a device used for measuring depth in the vertical direction. A SBES generates a single, narrow-angle acoustic pulse, which enables the recording of only depth data following sounding profiles [9]. The disadvantage of this measurement is the lack of information on the depths between the profiles. A measurement system built from a Global Navigation Satellite System (GNSS) and a SBES can be a separate system to record the X, Y, Z data synchronically in the Universal Transverse Mercator (UTM) system;
- A MultiBeam EchoSounder (MBES) is a system that records bathymetric data over a wide swath of the bottom, perpendicular to the direction of a vessel’s movement. The transducer in a multibeam echosounder generates multiple acoustic beams. For this reason, bathymetric surveys conducted using a MBES can completely cover the studied bottom with depth data [10]. Multibeam echosounders are applied in both shallow and deep-water surveys. The data derived from this system are expressed in the vertical datum;
- A SOund Navigation And Ranging (SONAR) is a device used to determine the location and classification of submerged objects using sound waves. A sonar emits sound pulses in water, which are then sent out and reflected off the bottom, fish or vegetation. The returning sound pulses are converted into electrical signals. The measured velocity of sound wave propagation in water enables both the estimation of the depth of the object from which the wave was reflected and the identification of underwater objects. Sonar data provide information on underwater objects that may pose a navigational hazard for other vessels.

Monitoring studies of the terrestrial part of the coastal zone apply optoelectronic methods that use the properties of light to record and process geospatial data. The optoelectronic measurements provide the necessary support to hydroacoustic surveys, as they allow data to be acquired from the marine coastal zone in shallow water areas and those adjacent to the coastline [11]. The operation of optoelectronic devices involves the conversion of electrical signals into optical signals and of optical signals into electrical signals. The following devices and systems are used to carry out the geodetic and hydrographic tasks in optoelectronic measurements (Figure 1):

- A positioning system determines the position coordinates of an aerial vehicle. An optoelectronic system can be equipped with a GNSS receiver which records curvilinear coordinates (B, L, h) in relation to the reference ellipsoid applied or in Cartesian coordinates (X, Y, Z). Where an optoelectronic device includes a low accuracy GNSS system, it is reasonable to determine the coordinates of selected points in the field using satellite techniques. Thanks to this method, it is possible to make a correction to the vehicle’s coordinates;
- An image sensor is used to convert electromagnetic waves into electrical impulses which are converted in the electronic system into an image of the scanned surface. In optoelectronic devices, an image is obtained using a so-called photodiode detector, a photomultiplier tube or Charge-Coupled Device (CCD) and Complementary Metal-Oxide-Semiconductor (CMOS) cameras. Image sensors are used in photo cameras, radars, sonars and Unmanned Aerial Vehicles (UAV) [12];
- An INS system is a device for measuring accelerations, rotation angles in three planes (X, Y, Z), and the Earth’s magnetic field. One of the components of the INS system installed on aircraft is an Inertial Measurement Unit (IMU), i.e., a device comprised of sensors such as accelerometers, gyroscopes and magnetometers. The system provides information on the vehicle’s motion parameters, i.e., the acceleration and velocity as well as the orientation in space [13]. The measurements of the IMU angles are taken in relation to the locally adopted coordinate system;
- A laser rangefinder is an instrument used to measure distance. A rangefinder sends out a laser pulse in the form of a laser beam, which is then reflected off the surface being measured and returns to the measurement instrument. The device circuit then calculates distances based on the time between the emission and reception of the sound wave. A rangefinder is a stand-alone instrument which is also available in the form of modules that are incorporated into considerably larger systems or integrated with additional systems, e.g., camera or a GNSS. Data logging is recorded in a local system;
- A Terrestrial Laser Scanning (TLS) is a system that measures the angle and distance between the instrument and the surface being measured. As a result of the reflection of a laser beam off the observed object, both the distance between the instrument and the measurement point, as well as the horizontal and vertical angle are determined. Surveys carried out using TLS scanning provide geospatial data on the studied object or surface in the form of a point cloud, recorded in the local system of the device with the coordinates of X, Y, Z [14];
- An Airborne Lidar Bathymetry (ALB) is a device that measures the distance from a flying aircraft to the ground points. The airborne laser scanning system includes three main devices, i.e., a laser rangefinder interfacing with the GNSS and INS systems. The data integration from these three measurement systems provides information on the position from which the distance measurement was taken and the distance itself and its direction in space. The device records the ground point coordinates in the rectangular coordinate system [15];
- A RAdio Detection And Ranging (RADAR) is an instrument that determines the angle, distance (range) or velocity of an object. The radar operation involves the measurement of the time between the signal transmission and the recording of its echo. During hydrographic surveys, the device supports automated measurement platforms in monitoring the environment while preventing hazardous events.

If bathymetric and topographic data are collected separately, it is complex to use them together due to differences in accuracies, datums and formats. Therefore, the data integration is essential. It is a process that involves collecting data from different sources and homogenising them in a database to provide a unified environment for modeling, processing and visualization [16]. The first step toward solving the problems of integration is a description and comparison of the methods used before.

The paper describes selected data integration methods important from the point of view of the authors of the review. This publication is structured as follows: The Introduction presents issues and motivation to discuss GNSS, hydroacoustic and optoelectronic data integration methods used in hydrography. It indicates the occurrence of spatial data of various types and is characterised by different coordinate systems. Chapter 2 contains a description of four data integration methods. The paper concludes with final (general) conclusions that summarise its content. In particular, the accuracy of selected GNSS, hydroacoustic and optoelectronic data integration methods used in hydrography was presented in this section.

## 2. Review of GNSS, Hydroacoustic and Optoelectronic Data Integration Methods

### 2.1. Method of GNSS, TLS, UAV and USV Data Integration according to Dąbrowski P.S. et al.

**Figure 2:**
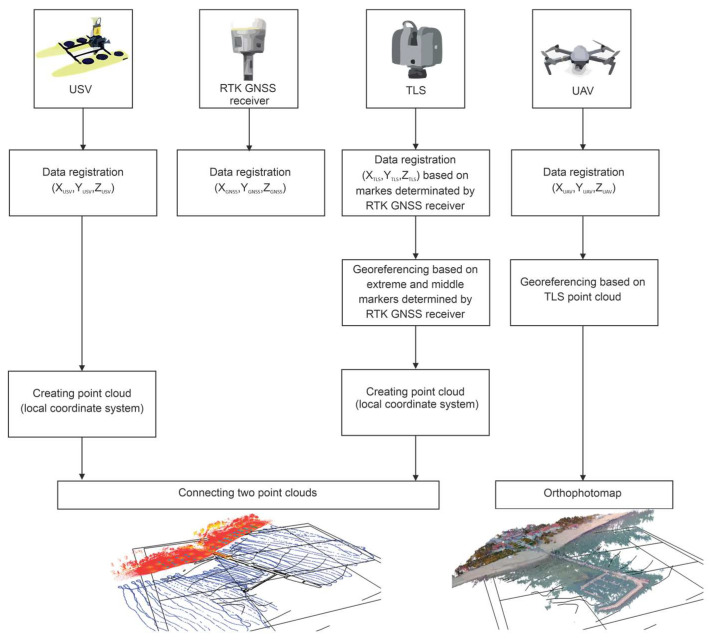
A simplified block diagram presenting the GNSS, TLS, UAV and USV data integration according to [17].

The method of data integration [17] was developed based on the tombolo phenomenon measurement campaign in Sopot in 2019, during which land GNSS measurements, laser scanning, hydrographic [18] and photogrammetric [19] surveys were performed. The authors of the article noted the indeterminacy problem of geodetic and hydrographic coordinate systems in the data integration, and they accurately described mathematical procedures to bring the data to a uniform reference system and then verify the data on the measurement results from the campaign. The presented actions are necessary when integrating data derived from different sources. Figure 2 provides a simplified block diagram presenting the GNSS, TLS, UAV and Unmanned Surface Vehicle (USV) data integration. Further on in the method analysis, the major aspects of data harmonisation are described.

The harmonisation process of a three-dimensional dataset [20] involves the determination of the scale factor, three rotation angles around three axes of local coordinate systems and the translation vector while considering the height coordinates of three-dimensional sets in the transformation. In three-dimensional space, rotations around the axes are described using elementary rotation matrices [21], which are functions of rotation angles around selected axes of coordinate systems [22]. The formula for the harmonisation of three-dimensional data devoid of vertical deviations was implemented based on the following formulas [17]:

(1) $$x\prime =x\prime \prime \cos\left(\theta \right)-y\prime \prime \sin\left(\theta \right)+{\vec{T}}_{X},$$

(2) $$y\prime =x\prime \prime \sin\left(\theta \right)+y\prime \prime \cos\left(\theta \right)+{\vec{T}}_{Y},$$

(3) $$z\prime =z\prime \prime +{\vec{T}}_{Z},$$

where:

x′,y′,z′—point coordinates in the local based coordinate system (X′,Y′,Z′);
x″,y″,z″—point coordinates in the local modified coordinate system (X″,Y″,Z″);
θ—rotation angle;
${\vec{T}}_{X},{\vec{T}}_{Y},{\vec{T}}_{Z}$—three-dimensional coordinates of the translation vector.

However, the formula for the harmonisation of spatial data with a found deviation in their numerical representation from the vertical is written as follows:

(4) $$R=U\cdot \Lambda \cdot V^{T},$$

where:

R—rotation matrix;
$U,V^{T}$—partial rotation matrices;
Λ—scaling matrix.

Before undertaking the data harmonisation process, it was decided that the target system would be the PL-2000 plane coordinate system, while the elevations would be expressed in the normal height system. The first stage of work involved the data georeferencing [23] derived from TLS, based on the presented mathematical assumptions. In the first place, a TLS point cloud was recorded in an unspecified local coordinate system. The scans were then combined into a single point cloud and georeferenced in relation to the extreme and middle markers obtained from land GNSS measurements. The next stage of work involved the determination of the scale change coefficient in relation to the TLS point cloud and the reference GNSS RTK surveys. On the other hand, the rotation matrix and the translation vector were calculated. The characteristic point coordinates were then compared to the values derived from land GNSS measurements.

The next spatial data set, whose coordinate values were reduced to the values of PL-2000 plane system’s coordinates and the normal height system, was derived from UAV surveys. The obtained point cloud generated from the photogrammetric model was initially georeferenced, but its high inaccuracy necessitated the correction of the point locations. Therefore, the TLS cloud was adopted as a reference object in relation to the UAV cloud. At this stage, the main aim was to determine the transformation parameters from both clouds (TLS and UAV), from which control points were later determined. At the data processing stage, a scale difference was noted in the spatial sets of both clouds. Therefore, the Singular Value Decomposition (SVD) method was applied to obtain the components of rotation matrices, from which in the next step the rotation angles around the X, Y, Z axes were determined. The last stage was the spatial rotation operation taking into account the V_OFF_ vector is expressed by the following formula:

(5) $$P^{I}=U\cdot \Lambda \cdot V^{T}\cdot \left(P^{\mathrm{II}}+V_{\mathrm{OFF}}\right)-V_{\mathrm{OFF}},$$

where:

$P^{I}$—adjustment point coordinates in the corrected coordinate system;
$P^{\mathrm{II}}$—adjustment point coordinates in the corrected coordinate system after rotation;
$V_{\mathrm{OFF}}$—offset vector.

The bathymetric data were assigned the coordinates from land GNSS measurements, and the depths recorded by the echosounder were obtained in the target coordinate system. The transformations performed on the actual spatial data confirmed the effectiveness of the mathematical procedures used in the spatial data harmonisation process. The integration of data from GNSS, TLS, UAV and USV surveys (Figure 2) indicates the significance of the data harmonisation process. An important aspect is to acquire and identify the coordinates of the corresponding points in datasets with different coordinate systems. Georeferencing is a necessary component for data integration, particularly when these data are derived from different measurement instruments. The material, presented in the form of an article, is a valuable source of information on procedures for the data transformation derived from hydroacoustic (USV) and optoelectronic (TLS and UAV) systems [17].

### 2.2. Method of GNSS, TLS, UAV and USV Data Integration according to Erena M. et al.

**Figure 3:**
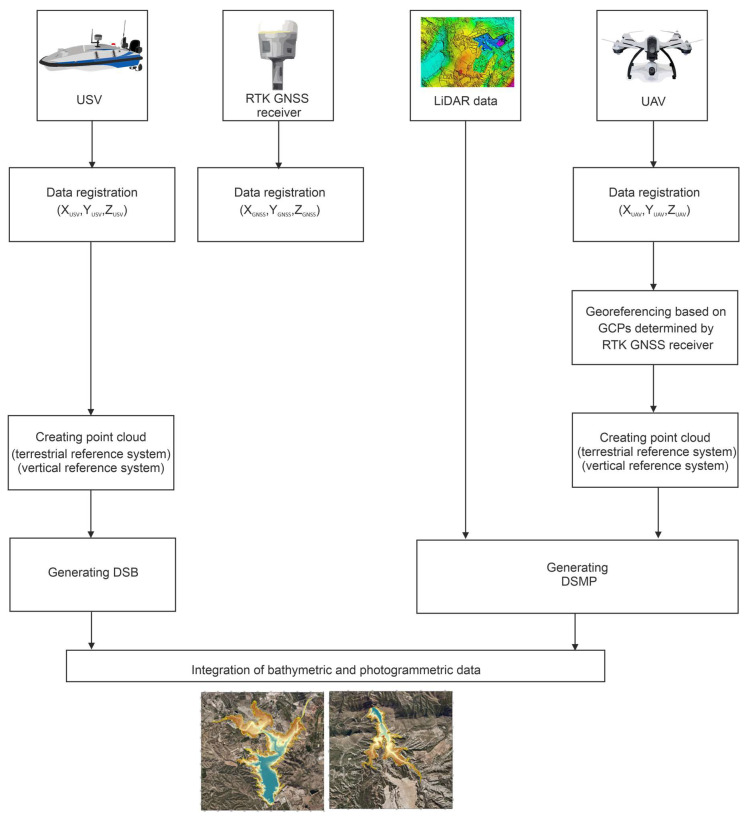
A simplified block diagram presenting the GNSS, TLS, UAV and USV data integration according to [24].

The data integration derived from hydroacoustic and optoelectronic systems is increasingly becoming a source of information on the changes taking place in the natural environment. An example of the use of data fusion in monitoring quantitative changes in water resources was a study conducted on composite data derived from hydroacoustic and optoelectronic measurements on the reservoirs of the Segura River Basin [24]. Due to climate change resulting in a reduction in the annual precipitation values, waterbodies in this region are becoming shallower [25]. Therefore, numerous measurement campaigns were conducted, including Aerial Laser Scanning (ALS), bathymetric sounding using an USV and a photogrammetric pass using two UAVs. Based on the data obtained from surveys, a model was then generated to provide information about the total capacity, the volumes of sedimented materials in the reservoirs and their water retention capacity. The presented data integration method [24] is based on the use of spatial information systems in the process of building spatial data models (Figure 3).

The main works when developing the topobathymetric model involved the recording and processing of data, as well as assigning them a specified coordinate system. In order to record both the surface area of the waterbody with the adjacent area and the waterbody volume, two low-altitude passes with a spatial resolution of 0.05 and 0.2 m, using a Sony QX1 photogrammetric camera, were made. However, when a high-altitude flight was required, the Sony camera was installed on a Cessna 150 aircraft. Additionally, a measurement of the coordinates of Ground Control Points (GCP) was taken by the Global Positioning System (GPS) RTK method [26], which enabled the transformation of the photograph coordinates, expressed in a pixel system (of the image), to the World Geodetic System 1984 (WGS-84). The photographs were then classified and matched while creating a uniform X, Y, Z point cloud. The generated point cloud was then transformed to the European Terrestrial Reference System 1989 (ETRS89) and the elevations were referred to the European Vertical Reference System 1989 (EVRS89). The prepared data were implemented into a geodatabase in the ArcGIS software in order to create, at a later stage, a terrain model along with the processed data derived from the Light Detection And Ranging (LiDAR) system. Moreover, the photogrammetric data were used to create a Digital Terrain Model (DTM) in the form of a Triangulated Irregular Network (TIN) [27] using the ArcGIS software. The processed photogrammetric data in the form of a TIN model enabled the data acquisition on the capacity of the studied reservoirs.

The next phase of work involved the development of bathymetric data derived from a GNSS RTK receiver, an USV equipped with an echosounder and a sonar. The integrated system recorded the vehicle’s position data in the ETRS89 system. The depths were recorded in the EVRS89 system in relation to the mean sea level in Alicante, pursuant to the INSPIRE Directive [20].

In quantitative analyses of waters, high accuracy elevation data (which are often acquired using the ALS method) are necessary. The ALB survey was conducted with a scanning density of 0.5 point/m^2^ and covered the area of the entire Segura River Basin. The height values, as for the depth, were recorded in the EVRS89 system in relation to the sea level in Alicante. LiDAR data were converted using ArcGIS 10.5 and LAStools softwares.

The process of bathymetric and optoelectronic data integration was carried out using the ArcGIS 10.5 software. The data derived from the USV were used to generate a Digital Surface Model Bathymetry (DSMB), while the data from the LiDAR and UAV helped acquire a Digital Surface Model Photogrammetry (DSMP). The photogrammetric data integration with the data acquired from laser scanning contributed to the improvement in the quality of the models obtained. The above models were combined with each other, thus creating a terrain model with a spatial resolution of 1 m. The implementation of the data integration process in the GIS is rather common [28,29]. However, it is the methods applied for acquiring spatial data that deserve special attention. For the purposes of the development of a DTM, tests were conducted using both hydroacoustic and optoelectronic systems. Additionally, the process of preparing data derived from different systems (which determines the accuracy of data integration) was discussed.

### 2.3. Method of UAV and USV Data Integration according to Genchi S.A. et al.

**Figure 4:**
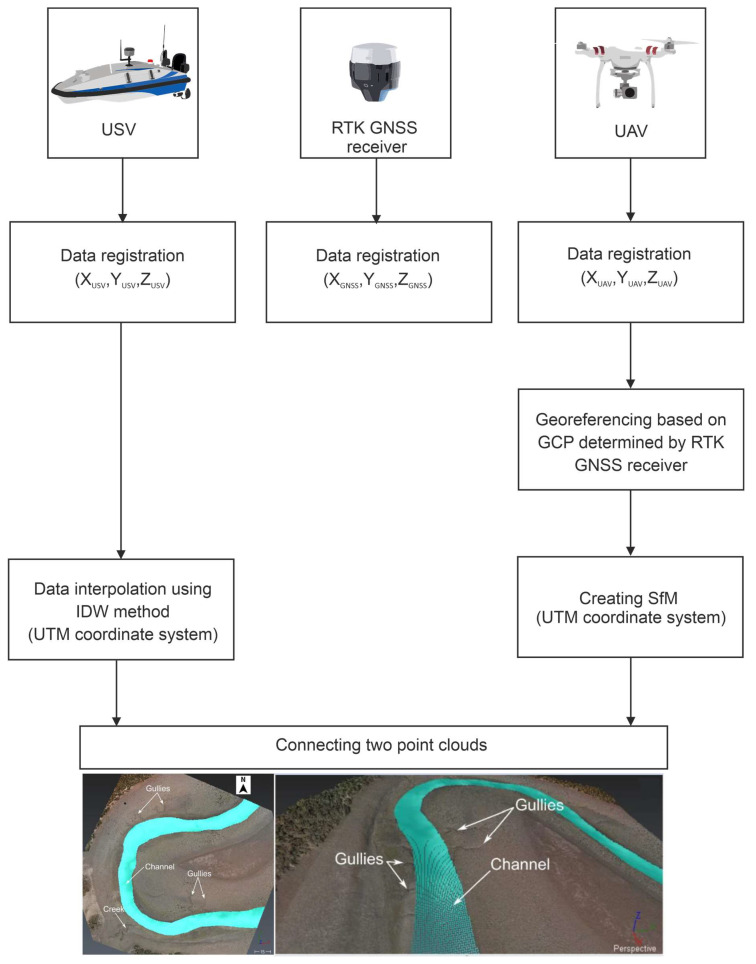
A simplified block diagram presenting the UAV and USV data integration according to [30].

The method of UAV and USV data integration [30] was developed based on UAV and USV measurements conducted in November 2018 and January 2019, respectively, in the Bahia Blanca Estuary. The estuary comprises a meandering tidal channel and tidal flats, i.e., a wide plain that is flooded at high tide [31]. It is a waterbody characterised by variable hydrodynamic conditions, affected by the tidal phenomenon. Figure 4 presents a diagram showing the main stages of the proposed UAV and USV data integration method.

One of the aims of the study was to generate a topographical terrain model that covers the river mouth area using the Structure from Motion (SfM) method [32]. This involved the creation of a three-dimensional model from a set of photographs, based on the observation and description of the location of points on the basis of a change in the perspective, i.e., the location of camera positions. To this end, a photogrammetric pass was conducted using a DJI Phantom 3 standard quadcopter. When planning the pass in the intertidal zone, the atmospheric conditions associated with tidal flows were taken into account. The UAV measurement was taken at low tide, as it enabled the recording of a larger terrain area. Moreover, before the pass, the coordinates of seven GCPs and four checkpoints were determined using a GNSS RTK receiver. The first stage of data processing involved image recording and matching. It was described in detail in [33]. Next, the georeferencing process was undertaken. The point cloud was assigned a spatial reference WGS-84/UTM zone 20S based on the coordinates of GCPs, while the SfM algorithm was used to generate a dense point cloud. Furthermore, the model accuracy was checked by comparing selected coordinates of the three-dimensional model with the coordinates of control and ground points [34]. The accuracy was assessed by determining the Root Mean Square Error (RMSE). The RMSE of the interpolated Topographic Point Cloud (TPC) in relation to the ground points amounted to: 0.13 m for the northern coordinate, 0.15 m for the eastern coordinate and 0.007 m for the height coordinate. The SfM method applied enabled the generation of a point cloud comparable, in terms of accuracy and density, with the data acquired by means of ALS and TLS. The last data processing stage involved the cleaning of data covering the aquatic and coastal areas. The performed operation allowed a three-dimensional model without water coverage to be obtained.

Bathymetric surveys were carried out using an USV equipped with a GPS receiver and a SBES. As with photogrammetric measurements, the atmospheric conditions were of importance. The bathymetric survey was conducted at high tide to record the depths and the spatial extent of water. Moreover, the depths obtained were referred to as the tidal height. The information on the tides was acquired from the nearest hydrometeorological station. The bathymetric data required no georeferencing, as they were assigned coordinates from a GNSS receiver. Since the Bathymetric Point Cloud (BCP) did not cover the entire area with data, it was reasonable to perform data interpolation. The data were processed using the most well-known interpolation methods: Inverse Distance Weighting (IDW) [35], kriging [36], minimum curvature and natural neighbour. Next, the accuracy of each interpolated BCP was assessed in relation to the TPC using typical accuracy measures: Mean Absolute Error (MAE) [37], RMSE [38] and coefficient of determination (R^2^):

(6) $$\mathrm{RMSE}=\sqrt{\overset{n}{\underset{i=1}{\sum }}\frac{{\left({\hat{y}}_{i}-y_{i}\right)}^{2}}{n}}$$

(7) $$\mathrm{MAE}=\frac{1}{n}\overset{n}{\underset{i=1}{\sum }}\left|{\hat{y}}_{i}-y_{i}\right|$$

(8) $$R^{2}=\frac{\sum_{i=1}^{n}{\left({\hat{y}}_{i}-\overset{¯}{y}\right)}^{2}}{\sum_{i=1}^{n}{\left(y_{i}-\bar{y}\right)}^{2}}$$

where:

n—number of points;
$y_{i}$—y value for observation i;
${\hat{y}}_{i}$—predicted value of y for observation i;
$\bar{y}$—arithmetic mean of y value.

According to the proposed accuracy assessment, the IDW method was selected. The RMSE value (0.18 m) of the interpolated BPC in relation to the TPC indicates a high degree of model fitting. The coefficient of determination also indicated a very high model fitting (0.90). On the other hand, the coordinates of the BPC deviate from the TPC by 0.05 on average (MAE).

**Figure 5:**
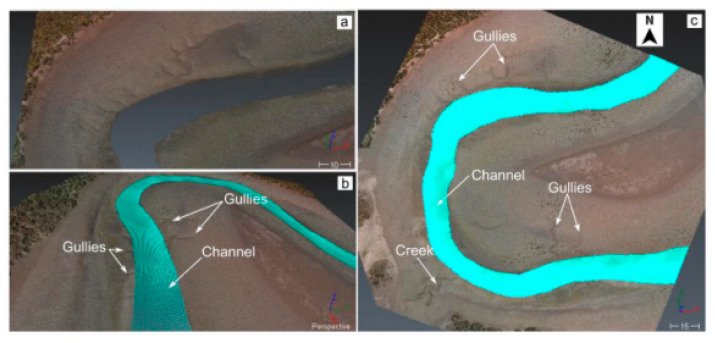
Different views of point clouds: (**a**) TPC; (**b**,**c**) topobathymetric point cloud presenting the combination of two data sources and its main terrain characteristics according to [30].

The final stage of work involved the integration of two-point clouds. In order to visualise the model, the overlapping points were removed from the BCP. The topobathymetric model (Figure 5) had a spatial resolution of 0.08 m for the topographic part and 0.5 m for the bathymetric part.

The topobathymetric model is distinguished by the mapping of such hydrological features as the bay, channel and gorges. This is certainly influenced by the area under study, and the SfM method applied. The advantage of the topobathymetric model comprising SfM is the possibility of visualising tidal conditions such as Mean High Water Neaps (MHWN), Mean High Water Springs (MHWS), Mean Low Water Neaps (MLWN), Mean Low Water Springs (MLWS) and Mean Sea Level (MSL) in the pre-determined area. The accuracy measures (MAE, R^2^ and RMSE) applied for the interpolated terrain model deserve special attention.

The topobathymetric model shows the dynamic conditions of the area (tide at different conditions/stages) and the topography. Therefore, the described example can be qualified as an attempt to use the data assimilation process [39] in data integration. Data assimilation based on the regional climate model or the surface model is a new issue that will contribute to the development of data integration methods in the future.

### 2.4. Method of LiDAR, NOAA and USGS Data Integration according to Gesch D. and Wilson R.

**Figure 6:**
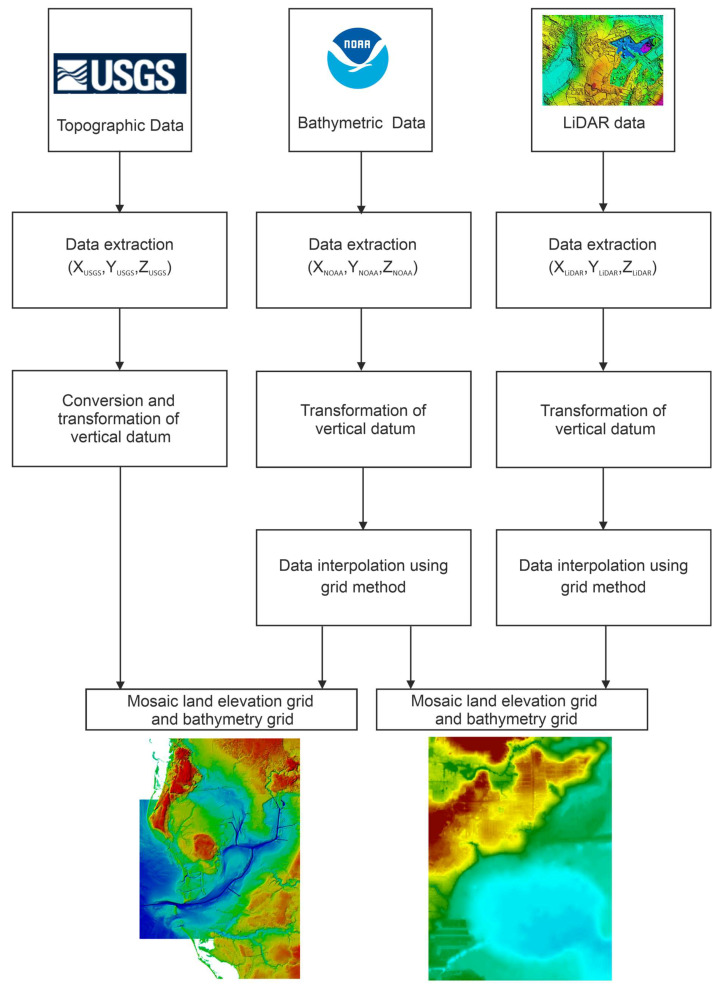
A simplified block diagram presenting the LiDAR, NOAA and USGS data integration according to [40].

One of the first DTMs, a topographic-bathymetric elevation model of the coastal zone, was created by integrating the data [40] covering the Tampa Bay area (Figure 6). In view of the spatial extent of the area, the data measured by the National Oceanic and Atmospheric Administration (NOAA) were used [41], while the topographic data was acquired from the United States Geological Survey (USGS) database. Moreover, LiDAR data were used for the first time when developing a topobathymetric model. The research work was initiated as part of the project aimed at developing techniques and tools to facilitate the integration of data derived from different sources [42].

A key component in the creation of a model is to prepare input data which must have a uniform reference system. The first stage of works commenced with the construction of an elevation database. To this end, the topographic data covering the bay area were extracted from the USGS National Elevation Dataset (NED). The NED comprised data derived from both optoelectronic measurements and topographic maps. They were converted to a horizontal reference North American Datum of 1983 (NAD 83) and the heights were expressed in relation to the North American Vertical Datum of 1988 (NAVD 88). Since the extracted topographic data were recorded in the target datum, the georeferencing process was not implemented. However, for the purpose of processing the elevation data, the height unit of a foot was assigned, and the data transformation to the reference NAVD 88 was carried out.

The bathymetric data were processed in several stages. In the first step, the depth data (approx. 800,000 X, Y, Z points) were imported to the ArcView software. They were derived from 47 bathymetric soundings conducted in the years 1950–1956. The data overlapped due to the extensive temporal frequency of surveys. As a result, a process was implemented to select the bathymetric data with the most recent date. The next stage involved the height transformation of the reference system. Depth points were initially recorded in relation to the vertical reference system of the Mean Low Water (MLW) and the vertical reference system of the Mean Lower Low Water (MLLW). In view of the above, the transformation was performed using the VDatum tool [43,44,45], which allowed the depth data to be assigned a NAVD 88 system. The final stage of the depth data preparation involved the generation of a DTM in the form of a GRID model.

A very important aspect of this integration was the combination of the data derived from LiDAR and the depth data for the first time. The topographic data derived from the aerial LiDAR conducted by the University of Florida were converted for several test areas to demonstrate both the usefulness of incorporating the most recent high-resolution data and the high accuracy of the topobathymetric model. As part of the test work, the X, Y, Z coordinates were recorded and assigned NAD 83 and NAVD 88 systems and a GRID model was generated.

Before undertaking the main stage of work, representative bathymetric points and elevation points from USGS were selected to determine the coastline, and on its basis, the models were fitted. It was necessary because the data did not overlap in terms of time. After determining the coastline course, the points selected from it were converted with the ArcInfo software using the Thin Plate Spline (TPS) algorithm [46]. The final step in data processing was the mosaicking of the bathymetric and elevation grids. The automated mosaicking technique is indicated for a large area. The mosaicking process involves the processing of a set of several or a few dozen digital component images to obtain a single image.

**Figure 7:**
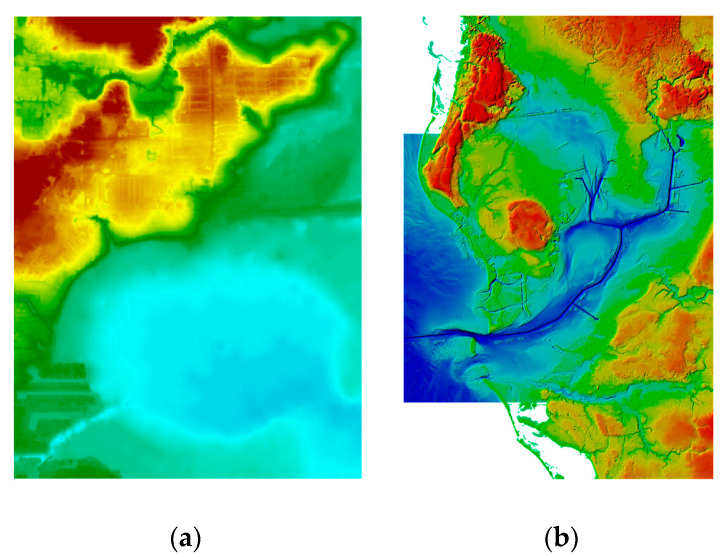
Results of the integration of (**a**) bathymetric and LiDAR data; (**b**) depth and topographic data according to [40].

The resulting model ensured complete coverage of the area. The procedure for generating the depth and LiDAR models was the same as for the bathymetric and topographic USGS data [47]. The integration of LiDAR and NOAA data enabled the creation of a high-resolution raster (Figure 7).

## 3. Discussion and Conclusions

Conducting complex spatial analyses of water areas requires an integrated approach to measurement and data integration. However, thus far no procedures have been developed to integrate hydrographic data from hydroacoustic and optoelectronic systems. The problem in processing a universal method of data integration probably results from the continuous development of measurement techniques, the presence of diverse spatial data with different coordinate systems and the specificity of the studied waterbody.

This paper is an attempt to analyse selected methods of data integration, which additionally presents the process of data acquisition and processing. The method review will contribute significantly to the development of the data integration model, which will be presented in the next publication.

The assessment of the applied mathematical assumptions in data integration [17] was verified on the basis of the characteristic point coordinates of the TLS cloud related to the PL-2000 plane coordinate system, which were compared to the coordinates obtained from land GNSS measurements. The deviation values did not exceed 0.016 m in the horizontal plane, however, the deviation values did not exceed 0.027 m in the vertical plane. This proves the accuracy of the harmonisation process. No verification procedures were performed for the GNSS, TLS, UAV and USV data integration methods [24]. In the case of data integration accuracy [30], the RMSE of the interpolated TPC in relation to the ground points amounted to 0.13 m for the northern coordinate, 0.15 m for the eastern coordinate and 0.007 m for the height coordinate. The accuracy of the point cloud generated by the SfM method is comparable with the data from LiDAR (accuracy at the level of 0.15–0.25 m). It is also worth paying attention to the procedure of selecting the best interpolation method for bathymetric data. The values of MAE, R^2^ and RMSE were calculated for the interpolated BCP with respect to the TPC. According to the proposed accuracy assessment, the IDW method was selected. The RMSE value (0.18 m) of the interpolated BPC in relation to the TPC indicates a high degree of model fitting. The coefficient of determination also indicated a very high model fitting (0.90). On the other hand, the integration of large-scale data [40] processed into two numerical models (BPC and TPC) was characterised by high accuracy. The accuracy was assessed by comparing the bathymetric model with precise reference data. As a result, the RMSE value of 0.43 m was obtained. The accuracy of selected GNSS, hydroacoustic and optoelectronic data integration methods used in hydrography is presented in Table 1.

According to the International Hydrographic Organization (IHO) S-44 standard [48], hydrographic surveys described in this paper should be carried out with respect to Order 1a. Data used in the method proposed by Gesch D. and Wilson R. come from many sources and vary in time, and therefore, they were initially classified as those that do not meet IHO requirements. In other methods, bathymetric measurements were not verified with indicators such as Total Propagated Uncertainty (TPU) and Total Vertical Uncertainty (TVU), as well as data did not have 100% bathymetric coverage. In summary, the presented methods do not meet IHO requirements.

The analysed examples show that there is no single data fusion scheme. This is due to the different specifications of the devices used, research aims and types of waterbodies. In all schemes, the data fusion was multi-stage and required the use of commercial software such as ArcGIS, CloudCompare and VDatum. The integration of hydroacoustic and optoelectronic data is a new issue that requires detailed study. An alternative to complex methods of spatial data integration are machine learning methods which, using artificial intelligence, automate the process of creating models [49]. The model is built on the training set, which are model patterns. One of the machine learning methods is Artificial Neural Networks (ANNs), which are used to transform coordinates with a small number of references or to create DTMs. Machine learning methods will help to improve the accuracy of integrated data, assuming that the training set and the analysed data will come from the same devices and systems [50]. In addition, in the future, an integral system should be created in which data processing would take place at the stage of obtaining data.

## Figures and Tables

**Table 1:** Accuracy of selected GNSS, hydroacoustic and optoelectronic data integration methods used in hydrography.

Measurement Accuracy	Method According to Dąbrowski P.S. et al.	Method According to Genchi S.A. et al.	Method According to Gesch D. and Wilson R.
dN ^1^	0.023 m	–	–
dE ^2^	0.16 m	–	–
dNH ^3^	0.027 m	–	–
RMSEx ^4^	–	0.15 m	–
RMSEy ^5^	–	0.18 m	–
RMSEz ^6^	–	0.007 m	–
RMSE ^7^	–	0.18 m	–
MAE ^8^	–	0.05 m	–
R^2 9^	–	0.90	–
RMSE ^10^	–	–	0.43 m

Maximum difference in Northing ^1^, Easting ^2^ and normal height ^3^ coordinates with respect to reference points. RMSE of x ^4^, y ^5^ and z ^6^ coordinates with respect to the SfM model and 7 GCPs. RMSE ^7^, MAE ^8^ and R^2 9^ with respect to the BPC and TPC models. RMSE ^10^ with respect to the bathymetric grid and reference transect data.

## Data Availability

Not applicable.
